# Assessing antibody decline after chemotherapy of early chronic Chagas disease patients

**DOI:** 10.1186/s13071-021-05040-6

**Published:** 2021-10-20

**Authors:** Niamh Murphy, M. Victoria Cardinal, Tapan Bhattacharyya, Gustavo F. Enriquez, Natalia P. Macchiaverna, Alejandra Alvedro, Héctor Freilij, Pablo Martinez de Salazar, Israel Molina, Pascal Mertens, Quentin Gilleman, Ricardo E. Gürtler, Michael A. Miles

**Affiliations:** 1 Faculty of Infectious and Tropical Diseases, London School of Hygiene and Tropical Medicine, London, UK; 2 Facultad de Ciencias Exactas y Naturales, Laboratorio de Eco-Epidemiología, Universidad de Buenos Aires, Ciudad Universitaria, Av. Int. Güiraldes 2180, C1428EHA Buenos Aires, Argentina; 3 CONICET-Universidad de Buenos Aires, Instituto de Ecología, Genética y Evolución de Buenos Aires (IEGEBA), Buenos Aires, Argentina; 4 Hopital de Niños “Dr. Ricardo Gutiérrez”, CABA, Argentina; 5 Barcelona Institute for Global Health (IS Global), Barcelona, Spain; 6 Coris BioConcept, Gembloux, Belgium

**Keywords:** *Trypanosoma cruzi*, Chagas disease, Serology, ELISA, Rapid diagnostic test, IgG, IgG1, Pre-treatment, Post-treatment

## Abstract

**Background:**

Chagas disease remains a significant public health problem in Latin America. There are only two chemotherapy drugs, nifurtimox and benznidazole, and both may have severe side effects. After complete chemotherapy of acute cases, seropositive diagnosis may revert to negative. However, there are no definitive parasitological or serological biomarkers of cure.

**Methods:**

Following a pilot study with seven Bolivian migrants to Spain, we tested 71 serum samples from chronic patients (mean age 12.6 years) inhabiting the Argentine Chaco region. Benznidazole chemotherapy (5–8 mg/kg day, twice daily for 60 days) was administered during 2011–2016. Subsequently, pre-and post-chemotherapy serum samples were analysed in pairs by IgG1 and IgG ELISA using two different antigens and Chagas Sero K-SeT rapid diagnostic tests (RDT). Molecular diagnosis by kDNA-PCR was applied to post-treatment samples.

**Results:**

Pilot data demonstrated IgG1 antibody decline in three of seven patients from Bolivia 1 year post-treatment. All Argentine patients in 2017 (averaging 5 years post-treatment), except one, were positive by conventional serology. All were kDNA-PCR-negative. Most (91.5%) pre-treatment samples were positive by the Chagas Sero K-SeT RDT, confirming the predominance of TcII/V/VI. IgG1 and IgG of Argentine patients showed significant decline in antibody titres post-chemotherapy, with either lysate (IgG, *P* = 0.0001, IgG1, *P* = 0.0001) or TcII/V/VI peptide antigen (IgG, *P* = 0.0001, IgG1, *P* = 0.0001). IgG1 decline was more discriminative than IgG. Antibody decline after treatment was also detected by the RDT. Incomplete treatment was associated with high IgG1 post-treatment titres against lysate (*P* = 0.013), as were IgG post-treatment titres to TcII/V/VI peptide (*P* = 0.0001). High pre-treatment IgG1 with lysate was associated with Qom ethnicity (*P* = 0.045). No associations were found between gender, age, body mass index and pre- or post-treatment antibody titres.

**Conclusions:**

We show that following chemotherapy of early chronic Chagas disease, significant decline in IgG1 antibody suggests cure, whereas sustained or increased IgG1 is a potential indicator of treatment failure. Due to restricted sensitivity, IgG1 should not be used as a diagnostic marker but has promise, with further development, as a biomarker of cure.

**Graphical abstract:**

We show that following chemotherapy of early chronic Chagas disease, a significant decline in IgG1 antibody suggests cure, whereas sustained or increased IgG1 is a potential indicator of treatment failure. Due to restricted sensitivity, IgG1 should not be used as a diagnostic marker but has promise, with further development, as a biomarker of cure.

**Figure:**
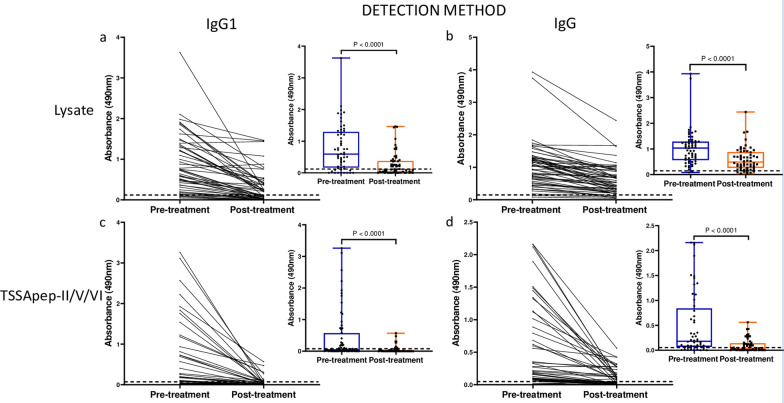

**Supplementary Information:**

The online version contains supplementary material available at 10.1186/s13071-021-05040-6.

## Background

Chagas disease, caused by the protozoan *Trypanosoma cruzi*, remains a major cause of disability in the Americas, particularly in the Gran Chaco region of Argentina, Bolivia and Paraguay. *Trypanosoma cruzi* is primarily transmitted via infected faeces of the triatomine bug vector, during a blood meal, when the parasite can enter the host through mucosal membranes and abraded skin. Transmission may also be congenital, by blood or organ donation, and orally via triatomine contamination of food or drink [1]. The initial acute phase of Chagas disease is often asymptomatic or without specific symptoms, although fatalities may occur [2]. The subsequent chronic phase may develop years later, in about 30% of individuals, principally with cardiomyopathy, and/or megasyndromes of the oesophagus and colon [3]. Infection can be cleared by a full course of chemotherapy with benznidazole (or nifurtimox). However, both drugs require prolonged treatment (30–60 days), and can be interrupted by severe adverse effects, particularly in adults. Delivery of chemotherapy has gained renewed impetus in the last 10 years, and treatment is now more accessible to rural communities [4–7] and urban centres [8]. However, the potential for improving long-term prognosis and for controlling transmission is usually lost due to the lack of early diagnosis and treatment, and delay in delivering insecticide control of infested dwellings, respectively [9].

Serological techniques to identify anti-*T. cruzi* immunoglobulin G (IgG), which are used principally in the chronic phase when parasites are sequestered in the tissues and rare in the circulating blood [10], include the enzyme-linked immunosorbent assay (ELISA), indirect haemagglutination (IHA), indirect immunofluorescence (IIF) and several commercial rapid diagnostic tests (RDTs), among the most commonly employed [10–12]. However, tests vary in practicality, sensitivity and specificity, and can be discordant between patients from different geographical locations [13]. During the chronic phase, other diagnostic techniques can be used, including molecular methods, for example kDNA-PCR, which amplifies sequences in the *T. cruzi* kinetoplast, a dense network structure of repetitive mitochondrial DNA, but these methods may lack sensitivity due to the paucity of circulating parasites. Therefore, serological identification of *T. cruzi*-specific IgG antibodies is considered the standard. However, the World Health Organization recommends at least two tests using different methods and/or detecting antibodies to different antigens and potentially a third test if the results are conflicting, in order to make a definitive diagnosis [14, 15]. Many biomarkers have been assayed as criteria of cure; however, reversion of conventional serology from positive to negative is considered the best and most reliable indicator of successful parasitological cure [14]. Nevertheless, except in treatment of initial acute cases or in the chronic phase during early childhood, the decline of conventional antibody (IgG) titres may take many years [16, 17], and patients therefore remain without confirmation of treatment outcome. Not having a definitive answer soon after chemotherapy is a fundamental impediment that can complicate patient management, and patients may be unwilling to start prolonged drug treatment if there is a risk of adverse side effects, with uncertain improvement of clinical prognosis, such as prevention of cardiomyopathy [18, 19]. Furthermore, with increased national and international migration, long-term patient follow-up is proving difficult and impractical. Thus, an early biomarker of cure is urgently needed [20].

*Trypanosoma cruzi* is composed of six genetic lineages or discrete typing units (DTUs), TcI–VI [21], with a possible seventh, TcBat [22]. TcI, TcII, TcV and TcVI are the most common in human infections, whilst TcIII and TcIV are principally associated with sylvatic cycles. It has long been proposed that the differing lineages may contribute to the varying clinical forms of Chagas disease throughout South America [23].

Various *T. cruzi* antigens or antigenic fractions that elicit a serological response have been evaluated for post-treatment biomarkers [24–26], with relative success [24]. The MultiCruzi assay, a serological assay incorporating 15 *T. cruzi*-specific antigens, when used with an interpretation formula, has been proposed for use as a predictive tool to assess parasitological cure in infants and children [27]. In paediatric cases post-chemotherapy, antibody titres to the trypomastigote small surface antigen (TSSA) shared by TcII, TcV and TcVI (TSSA-II/V/VI) decreased significantly faster than those against crude parasite homogenates [28]. Serology with a synthetic peptide TSSApep-II/V/VI epitope also revealed an association between seropositivity and severity of chagasic cardiomyopathy [29], and ELISAs and RDTs with protein G detection had the capacity to resolve host, ecological and epidemiological associations in the Argentine Chaco [30].

IgG is the most common class of immunoglobulin in human serum, the major antibody of the secondary immune response and is split into four subclasses, IgG1, IgG2, IgG3 and IgG4. IgG1 is at the highest levels in adult sera, with a relative abundance of 60% [31]. In comparison, although children are born with a relatively high level of IgG1 from the mother, this quickly drops to low levels due to non-sustained antigenic stimulation. From 6 months of age, the level of IgG1 increases, and by 5 years of age the level of IgG1 is 75% of that found in adult sera [32].

During the acute stage of infection, IgG is found at relatively low levels, and IgM is the most abundant antibody; however, as the infection changes from acute to chronic, there is a switch to IgG [33]. Of the four subclasses of IgG, IgG1, IgG2 and IgG3 are found at high titres during *T. cruzi* infection, with IgG1 being the most abundant, whereas IgG4 is at relatively low levels [34, 35]. Increased anti-*T. cruzi* IgG1 titres have also been associated with increased severity of Chagas disease cardiomyopathy [33]. Here we address whether IgG1 may be an early biomarker of cure after treatment of chronic Chagas disease. Following a pilot study, we assess IgG and IgG1 antibody decline in treated early chronic Chagas disease patients living in the Argentine Chaco where domestic transmission was interrupted, using separately whole cell lysate and TSSApep-II/V/VI antigens, and we show that IgG1 is more discriminative as a biomarker for assessing cure than IgG, irrespective of antigen.

## Methods

### Pilot serological study

For a pilot cohort of chronic patients (*n* = 7, all of Bolivian origin) presenting in Barcelona, Spain, serum samples at 0, 60 and 365 days (post-treatment) were assayed for anti-*T. cruzi* IgG and IgG1 levels by ELISA against *T. cruzi* lysate (described below). The data from these preliminary assays informed and encouraged the wider investigation in the main study.

## Main study

### Sample collection, serological surveillance and treatment in Argentina

Field work took place in the rural area of Pampa del Indio, Chaco Province, in northern Argentina [36]. In this municipality, intense domestic transmission of *T. cruzi* occurred and was suppressed by sustained actions against *Triatoma infestans,* the main vector, as part of an ongoing intervention programme launched in 2007, which virtually eliminated domestic infestation by the second year post-interventions [37–40]. During 2010–2016, we scaled up delivery of serodiagnosis and chemotherapy of seropositive people in the rural area, divided for operational reasons (areas I–IV) and achieved approximately 50% of serodiagnosis coverage of nearly 9000 inhabitants (Additional file 1: Figure S1). Patients included members of the Qom and Creole ethnic groups. The seroepidemiology and long-term impact of sustained vector control on domestic transmission are described elsewhere [40–43].

All serum samples were preserved at −20 °C until assayed for *T. cruzi* infection by conventional serology. In Argentina, the WHO guidelines for conventional serology are followed: the use of two serological tests, either ELISA, or IHA of IIF, detecting different parasite antigens or whole parasites [14, 15]. Here, duplicate ELISAs with non-recombinant (Chagastest, Wiener) and recombinant antigens (ELISA Rec v3.0, Wiener) were used, according to the manufacturer’s instructions. A serum sample was considered seropositive if reactive in two different assays [36]. Two serologically discordant human samples were sent to the National Institute of Parasitology “Dr. Mario Fatala Chabén” (ANLIS-Malbrán, Buenos Aires, Argentina) for final serodiagnosis, where they were tested by IHA, ELISA and IIF.

Benznidazole (5–8 mg/kg day) was administered twice daily for 60 days to all seropositive individuals, except in 2012 when nifurtimox was used (8–10 mg/kg day) (Additional file 1: Figure S1). Chemotherapy rounds were launched between 2011 and 2016. For the cohort treated in 2011, blood samples by venipuncture were taken by local physicians at day 0, 20–30, 60 and 180 post-treatment. Conventional serological tests were performed, and molecular diagnosis was applied by qPCR and kDNA-PCR to determine the infection status of the patients in the cohort treated in 2011 [36]. For this cohort, pre-treatment samples were collected between November 2010 and January 2011. The subsequent post-treatment samples were collected, as described above, when patients were resampled in 2017 in house-to-house visits.

### Molecular assays

For DNA extraction, samples were mixed immediately after collection with an equal volume of 6 M guanidine hydrochloride and 0.2 M EDTA in pH 8.0 buffer. Guanidine/EDTA blood samples were heated in a boiling water bath for 15 min. Total DNA was purified using a DNeasy Blood & Tissue Kit (Qiagen, USA) according to manufacturer’s instructions, slightly modified to exclude proteinase K and buffer AL [44] and eluted in 200 µl of distilled water. A total of 24 out of 34 pre-treatment Argentinean samples were positive by kDNA-PCR [36].

### Chagas Sero K-SeT RDT

As previously described [29, 30], the Chagas Sero K-Set is a novel RDT manufactured by Coris BioConcept using TSSApep-II/V/VI (GTENKPATGEAPSQPG) as the antigen and colloidal gold-conjugated protein G detection of bound IgG. Each test was assessed visually and independently by two individuals after 15 min of incubation. A test was considered valid if the control line was present, determined as positive if there was a signal of any intensity at the test (antigen) line, and negative if there was no signal at the test line. The intensity of signal at the test band was assessed visually as strong, weak or absent.

### ELISA

Immulon 4HBX 96-well flat-bottom ELISA plates (735–0465, VWR, UK) were divided into 16 sections of 3 columns × 2 rows of wells, to allow simultaneous assay of IgG1 and IgG paired pre- and post-treatment samples, as further described below. Wells were coated either with 100 μl/well of 1 × coating buffer (15 mM Na_2_CO_3_, 34 mM NaHCO_3_, pH 9.6) as a no-antigen control or with TSSApep-II/V/VI synthetic peptide (5 μg/ml) or lysate of *T. cruzi* TcI strain ISAN/US/00/Florida (2 μg/ml, prepared as described in [45]) diluted in 1× coating buffer. Following overnight incubation at 4 °C, plates were washed three times with PBS/0.05% Tween (PBST), then blocked with 200 µl/well of PBS/2% skimmed milk powder at 37 °C for 2 h. After three washes, 100 µl/well of serum at 1:200 dilution in PBST/2% milk was applied, such that for each paired sample per plate section, the upper row contained pre-treatment serum, and the lower row post-treatment serum. Following incubation at 37 °C for 1 h and six washes in PBST, 100 μl/well of horseradish peroxidase-labelled anti-human IgG (709–035-149, Jackson ImmunoResearch, USA) or anti-human IgG1 (ab99774, Abcam, UK), diluted 1:5000 in PBST/2% milk, were added to the second and third columns of each section, respectively. The first column of each section received PBST/2% milk only. After 1 h of incubation and six PBST washes, 100 μl/well of substrate solution (50 mM phosphate/citrate buffer, pH 5.0) containing 2 mM *o*-phenylenediamine HCl (P1526, Sigma-Aldrich) and 0.009% H_2_O_2_ (216,763, Sigma-Aldrich) was added to the entire plate, which was then incubated in the dark. Reactions were stopped with 50 μl/well of 2 M H_2_SO_4_, and absorbance values were measured at 490 nm. Duplicate (replica) plates were performed simultaneously, and mean results obtained. Cut-off values were calculated by subtracting the plate background absorbance values from each of the samples, including the negative controls; samples that were higher than the mean of the negative controls plus three standard deviations were considered positive. Only Chagas Sero K-Set RDT-reactive samples were assayed with TSSApep-II/V/VI ELISA; other lineage-specific RDTs are not yet available [30, 46].

### Data analysis

Two-tailed paired *t*-tests (pre- and post-treatment samples), unpaired two-sample *t*-tests or one-way ANOVA were used to determine statistical significance (GraphPad Prism, 8.4.3, San Diego, CA, USA). Values of *P* < 0.05 were considered statistically significant.

A serum sample was considered to decline significantly (herein called “clear decline”) if absorbance dropped by 50% (ELISAs) or exhibited a prominent decrease in RDT test line intensity (Additional file 2: Figure S2). For a given assay, a reactive serum pre-treatment and non-reactive post-treatment was considered seronegativisation. Re-examined patients (*n* = 71) were grouped according to whether they completed the 60 days of chemotherapy. Thirteen of the 71 patients reporting interruption of therapy [36] were designated as group B (age range, 2–20 years), and those with completed treatment [36] or self-reporting completion as group A (age range, 6–19 years), including the patient with therapeutic failure [36]. We compared the percentage of patients showing clear decline of antibodies or seronegativisation between groups by means of Fisher’s exact tests. Group B patients were excluded from IgG and IgG1 response pre-/post-treatment, but group A and group B were compared in the univariate analysis. We merged our database with that previously published [36], updating the data for corresponding patients, to investigate the association of antibody titres with individual patient data (i.e., age, body mass index [BMI], ethnic group). The relationship between antibody decline (for each serological test) and age at treatment, treatment groups and time elapsed since treatment (in years) was tested by multiple logistic regressions implemented in R using “lme4” and “sjPlot” packages [47–49]. Continuous variables were standardised.

## Results

### Pilot study shows trend of IgG1 decline in paired pre- and post-treatment sera

**Fig. 1:**
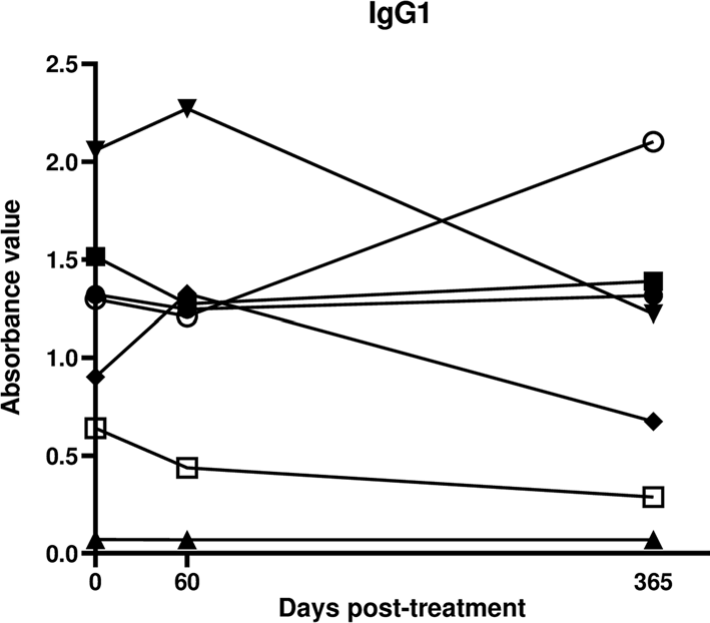
Pilot study supports IgG1 decline post-treatment: ELISA absorbance values are shown for individual participants at 0, 60 and 365 days post-treatment. Each line represents an individual patient. Three of seven show a decline in IgG1

To assess the trend of IgG subclass titres post-chemotherapy, sequential serum samples from seven adult Bolivian patients living in Barcelona were assayed by ELISA against *T. cruzi* lysate at 0, 60 and 365 days post-treatment. IgG1 antibody titre declined in three (42.3%) participants 365 days post-treatment (Fig. 1), whilst there was no antibody decline for IgG2 or IgG4; one participant showed IgG3 antibody decline 365 days post-treatment (data not shown).

## Main study

### Conventional serology remains positive after chemotherapy

Conventional serological assays were used with all 71 samples collected in 2017; all except one remained consistently positive. People re-examined were on average 12.6 years of age when treated; 47.9% were female, 52.1% were Qom descendants, and on average 5.0 years had elapsed since treatment. Around a third (34%) of the study people had moved from their original household to a new residence within the municipality when revisited in 2017.

### Molecular assays

kDNA-PCR was carried out with post-treatment samples collected in 2017 to indicate presence of parasitaemia. The predicted 330-bp amplicons were produced with pre-treatment samples but not produced with post-treatment samples (data not shown). All of the 71 post-treatment samples were negative.

### TSSApep-II/V/VI seropositivity by Chagas Sero K-SeT RDT

All 71 pre-treatment samples were initially screened with this RDT to detect recognition of TSSApep-II/V/VI. Fifty-four of the 58 pre-treatment samples from group A and 11 of the 13 pre-treatment samples from group B were positive by this RDT (Table 1) giving an overall seropositivity of 91.5%.

**Table 1:** Changes in anti-*T. cruzi* seroreactivity according to assay and treatment group

Antigen	Number positive/examined (%) by detection assay					
	Protein G: Chagas Sero K-SeT	IgG1 ELISA		IgG ELISA		
	TSSApep-II/V/VI	Lysate	TSSApep-II/V/VI^a^	Lysate		TSSApep-II/V/VI^a^
Group A (*n* = 58)						
Positive (pre-treatment)	54/58 (93.1%)	46/58 (79.3%)^f^	22/53^b^ (41.5%)^f^	57/58 (98.3%)^g^		41/53^b^ (77.4%)^g^
Clear decline	25/54 (46.3%)	35/46 (76.1%)	22/22 (100%)	21/57 (36.8%)		34/41 (82.9%)^e^
Seronegativisation	11/54 (20.4%)	19/46 (41.3%)^h^	15/22 (68.2%)^i^	6/57 (10.5%)^h^		18/41 (43.9%)^i^
Remained seronegative^c^	4/58 (6.9%)	12/58 (20.7%)	30/53^d^ (56.6%)	1/58 (1.7%)		12/53^d^ (22.6%)
Group B (*n* = 13)						
Positive (pre-treatment)	11/13 (84.6%)	11/13 (84.6%)	7/10^b^ (70.0%)	13/13 (100%)		10/10^b^ (100%)
Clear decline	5/11 (45.5%)	6/11 (54.5%)	6/7 (85.7%)	6/13 (46.2%)		5/10 (50.0%)^e^
Seronegativisation	1/11 (9.1%)	2/11 (18.2%)	5/7 (71.4%)^j^	1/13 (7.7%)		2/10 (20.0%)^j^
Remained seronegative^c^	2/13 (15.4%)	2/13 (15.4%)	3/10 (30.0%)	0/13 (0%)		0/10 (0%)

Group A: completed treatment; group B: reported interruption of treatment

^a^Only assayed for samples positive by TSSApep-II/V/VI Chagas Sero K-SeT RDT

^b^Two RDT-positive samples (one each from group A and group B) were not assayed by TSSApep-II/V/VI-ELISA

^c^Remained seronegative = negative pre- and post-treatment

^d^One sample seroconverted (changed from negative to positive) in this assay

^e–j^Statistically significant differences between superscript pairs are discussed in the text

### Comparison between treatment groups

We found a significantly higher percentage of patients showing a clear decline of antibody titres after treatment in group A than in group B when assayed against TSSApep-II/V/VI ELISA with IgG (Fisher’s exact test, *P* = 0.04; OR = 0.04; 95% CI = 1.07–23.33). We found no significant between-group differences in the percentage of positive patients before treatment and in those who became seronegative after treatment (Fisher’s exact test, *P* ≥ 0.1 in all cases) (Table 1).

### Chagas Sero K-SeT RDT is the least discriminative of the TSSApep-II/V/VI assays

Using the Chagas Sero K-SeT RDT in patients from group A, 25/54 (46.3%) of samples showed a clear decrease of band intensity post-treatment, in comparison to 22/22 (100%) or 34/41 (82.9%) by TSSApep-II/V/VI ELISA with IgG1 or IgG, respectively (Table 1). A similar trend was observed in patients from group B.

### ELISA with IgG1 is more discriminative than IgG in assessing seronegativisation and decline in antibody levels

**Fig. 2:**
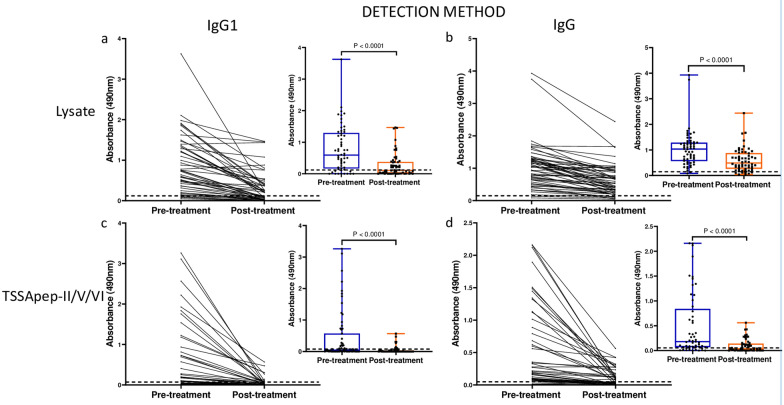
IgG1 decline is more discriminative than IgG in paired pre- and post-treatment sera for patients that completed treatment (group A). In each panel (**a**–**d**), ELISA absorbance values are shown connected for individual sample pairs, and in the insets as composite box and whisker plots, blue (pre-treatment) and red (post-treatment). **a** IgG1 with lysate (*P* < 0.0001 for pre- versus post-treatment); **b** IgG with lysate (*P* < 0.0001); **c** IgG1 with TSSApep-II/V/VI (*P* < 0.0001); **d** IgG with TSSApep-II/V/V (*P* < 0.0001). The dashed line represents each assay cut-off value

The sensitivities with pre-treatment samples were significantly greater against lysate than TSSApep-II/V/VI with both IgG1 and IgG (Fisher’s exact test, *P* < 0.0001; OR = 5.40; 95% CI = 2.31–11.91 for IgG1 and *P* = 0.001; OR = 16.68; 95% CI = 2.76–181.6 for IgG) (Table 1). However, the post-treatment decline in IgG1 was more discriminative than for IgG, regardless of antigen (Fig. 2). In group A patients, a clear decline in IgG1 was observed in 35/46 (76.1%) and 22/22 (100%) samples against lysate and TSSApep-II/V/VI, respectively, compared to 21/57 (36.8%) and 34/41 (82.9%) for IgG (Table 1). Overall, antibody titres also showed a greater decline with TSSApep-II/V/VI compared to lysate for IgG1 (paired *t*-test, *P* < 0.0001, *t*_(62)_ = 4.18 and *P* < 0.0001, *t*_(70)_ = 7.35, respectively; Fig. 2). Similarly, in group B patients, ELISA with IgG1 was more discriminative than with IgG (Table 1). The seronegativisation percentage was significantly higher with IgG1 regardless of the antigen employed (Fisher’s exact test, *P* < 0.0001; OR = 5.98; 95% CI = 2.26–17.70 for lysate and *P* = 0.057; OR = 2.74; 95% CI = 0.92–7.64 for TSSApep-II/V/VI) in Group A patients and for the TSSApep-II/V/VI in Group B patients (Fisher’s exact test, *P* = 0.052; OR = 10.00; 95% CI = 1.12–69.44) (Table 1).

In multiple logistic regression analysis, the occurrence of antibody decline (both IgG and IgG1) in ELISAs with lysate antigen decreased significantly with increasing age at treatment, but not with treatment group or time elapsed since treatment (Additional file 3: Table S1). Using TSSApep-II/V/VI as antigen, the occurrence of IgG antibody decline was significantly associated negatively with age at treatment and positively with treatment group. Seronegativisation was significantly negatively associated with both age at treatment and time elapsed since treatment (Additional file 4: Table S2). IgG1 antibodies assayed with TSSApep-II/V/VI were not included in this analysis, because virtually all samples declined.

### Univariate associations with serology

Univariate associations between IgG and IgG1 levels and lysate or TSSApep-II/V/VI ELISA are shown in Table 2.

**Table 2:** Univariate associations with IgG1 and IgG serology

ELISA antigen	Category	Categories	*N*	IgG Pre-treatment		IgG Post-treatment		IgG1 Pre-treatment		IgG1 Post-treatment	
				Mean absorbance value	*t*-test *P-*value	Mean absorbance value	*t*-test *P-*value	Mean absorbance value	*t*-test *P*-value	Mean absorbance value	*t*-test *P-*value
Lysate	Ethnic group	Creole	30	1.120	0.355	0.650	0.364	0.599	0.045*	0.244	0.529
		Qom	28	0.950		0.541		0.980		0.307	
	Completed treatment	Yes	58	1.038	0.540	0.597	0.209	0.783	0.079	0.274	0.013*
		No	13	1.159		0.778		1.180		0.589	
	Pre-treatment kDNA-PCR result	Positive	24	1.270	0.092	0.676	0.019*	0.910	0.156	0.121	0.281
		Negative	10	0.854		0.315		0.557		0.267	
TSSApep-II/V/VI	Ethnic group	Creole	26	0.700	0.030*	0.111	0.584	0.593	0.256	0.033	0.682
		Qom	27	0.321		0.091		0.327		0.046	
	Completed treatment	Yes	53	0.507	0.049*	0.101	< 0.0001***	0.478	0.660	0.038	0.261
		No	13	0.964		0.478		0.336		0.081	
	Pre-treatment kDNA-PCR result	Positive	21	0.738	0.640	0.105	0.996	0.591	0.194	0.0233	0.429
		Negative	9	0.592		0.105		0.172		0.012	

Unpaired *t*-test *P* values are shown

*P ≤ 0.05, **P < 0.01, ***P < 0.001

The Qom population had a significantly higher pre-treatment IgG1 titre against lysate. In contrast, the Creole population had a significantly higher pre-treatment IgG antibody titre against TSSApep-II/V/VI. In addition, there was a statistically significant association between patients not completing treatment and higher pre-treatment IgG titre against TSSApep-II/V/VI (*P* = 0.049, *t*_(61)_ = 2.01), higher post-treatment IgG titre against TSSApep-II/V/VI (*P* = 0.0001, *t*_(61)_ = 4.32) or higher 5-year post-treatment IgG1 titre against lysate (*P* = 0.013, *t*_(69)_ = 2.56), although titres were not significantly higher against TSSApep-II/V/VI. Furthermore, there was a significant association between a positive pre-treatment kDNA-PCR and higher post-treatment IgG antibodies against lysate (Table 2, *P*_(32)_ = 0.019, *t* = 2.46). No associations were found for the Chagas Sero K-Set RDT results pre- and post-treatment.

## Discussion

In Chagas disease, current methods to establish chemotherapeutic parasite clearance are technically demanding and inconclusive. Post-chemotherapy reversion of conventional serology from positive to negative may take decades to confirm parasitological cure. Failure to determine cure complicates patient follow-up, because physicians are unable to inform patients on their long-term prognosis and risk of developing chagasic cardiomyopathy or intestinal pathology. Furthermore, follow-up of patients may be difficult if they migrate to other regions or move to new households, and they may be unwilling to accept prolonged chemotherapy with potential adverse effects and no definitive outcome. Thus, there is a great need for identifying a biomarker for a rapid point-of-care test of cure. Molecular assays may be considered the most useful for assessing treatment response in the short term. However, there are no commercial molecular assay kits available, and typically only a fraction of seropositive chronic individuals are PCR-positive before treatment; moreover, a negative PCR cannot establish absence of infection, although a positive PCR proves treatment failure [50].

The role of the IgG subclasses as an early diagnostic tool, indicator of parasite clearance or predictor of disease prognosis has been assessed in other protozoan infections including malaria [51–54] and toxoplasmosis [55, 56]. In visceral leishmaniasis, the level of IgG1 response has been shown to be a potential therapeutic marker, principally in India; patients considered to be cured had significantly lower levels of anti-*Leishmania* IgG1 compared to those with treatment failure or relapse [57, 58], possibly due the lack of sustained antigenic stimulus associated with successful chemotherapy.

In a previous report of anti-*T. cruzi* IgG1 levels following accidental infection with *T. cruzi*, the anti-*T. cruzi* IgG1, IgG3 and IgG4 returned to pre-infection levels 55 days post-chemotherapy and 80 days after infection, whereas the IgG2 titres remained high at 300 days after infection [59]. Anti-*T. cruzi* IgG1 serology has been shown to be highly sensitive and specific for screening blood donors when conventional serological methods (ELISA and IIF) previously gave inconclusive results [60]. Similarly, anti-epimastigote IgG1 has been able to distinguish between *T. cruzi*-infected and non-infected individuals [61]. Most recently, the Human Chagas-Flow ATE-IgG1 has been able to differentiate between TcI, TcVI and TcII lineages with high accuracy [62].

**Fig. 3:**
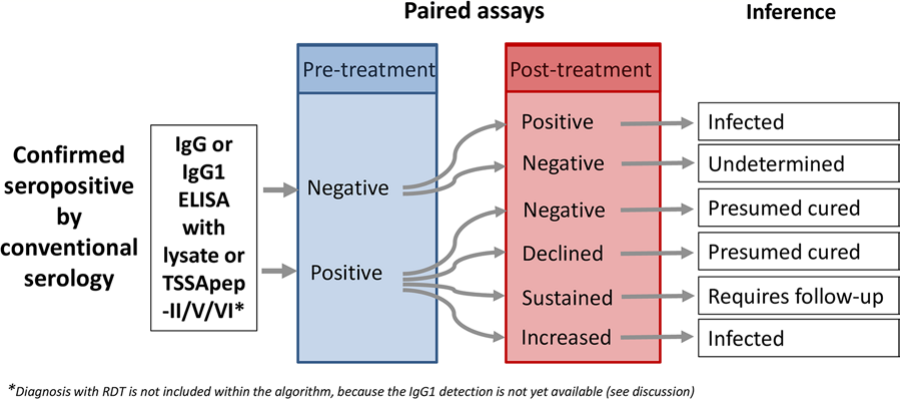
Suggested algorithm for chemotherapy follow-up after an average of 5 years post-treatment. Proposed pathway for incorporating IgG and IgG1 serology to infer chemotherapy outcome

Our pilot study of treated Bolivian patients residing in Barcelona initially detected a possible trend for a decrease in IgG1 titre compared to IgG and the other IgG subclasses. In the Argentine cohort of the main study, almost all patients remained seropositive post-treatment as determined by conventional serology, namely two commercial ELISAs that detect IgG with non-recombinant and recombinant antigens, showing that this could not determine whether treatment was successful. We assessed IgG and IgG1 antibody decline with ELISA against lysate and TSSApep-II/V/VI antigens pre- and post-treatment of early chronic Chagas disease patients. To our knowledge, there are few reports of TSSApep-II/V/VI as an antigen for assay with paired samples from chronic patients. Of pre-treatment samples screened by Chagas Sero K-SeT RDT for TSSApep-V/V/VI infection, 91.5% were positive, confirming that these are the predominant infecting lineages in the region, as previously reported [30, 63]. Among the patients who completed chemotherapy, a proportion of Chagas Sero K-SeT RDT positives (43.6%) showed IgG decline post-treatment, by visual assessment of band intensity. In contrast, IgG (82.9%) and IgG1 (100%) showed substantial decline by TSSApep-II/V/VI ELISA and even seronegativisation (Fig. 2). We propose an algorithm for incorporating IgG1 and IgG serological assays to infer the treatment outcome after chemotherapy when 5 years have elapsed (Fig. 3). In the ELISAs, IgG/IgG1 are detected with specific conjugates, whereas in the RDTs, antibodies are detected with protein G. Developing IgG1 RDTs could provide a useful tool for monitoring chemotherapy. Moreover, whereas the ELISA uses serum samples at a dilution of 1:200, RDTs use undiluted samples at the point of application, which may also explain the high RDT sensitivity with pre-treatment samples and the lower capacity to detect decline in antibody response. Modification of sample volume, together with specific IgG1 detection, could allow RDT assessment of antibody decline following chemotherapy (Fig. 3). Both antibody decline and seronegativisation were also observed in patients reporting an incomplete course of chemotherapy. Although the number of days of pill intake was missing for six out of 13 patients (group B), the remaining seven received an average of 20 days of treatment (range = 6–31) of the 60 days prescribed, thus on average less than half the treatment course. Shorter chemotherapy courses are now being trialed to avoid adverse reactions while sustaining parasiticidal effects [64, 65]. Similarly, all patients, including the 13 with incomplete treatment, had a negative kDNA-PCR post-treatment, suggesting that treatment may be effective with reduced length of chemotherapy. However, kDNA-PCR may have low sensitivity, and may not yield the correct diagnosis if there is a low number of circulating parasites.

We considered whether treatment completion, ethnic group, pre-treatment kDNA-PCR result, gender, age and BMI were univariate factors associated with IgG1 or IgG levels. Significant associations were found between post-treatment high IgG1 and failure to complete treatment. High IgG1 has been associated with greater deterioration of cardiac function in Chagas disease patients [35]. Interestingly, a significant association was found between lower levels of IgG post-treatment and a negative pre-treatment kDNA-PCR. We speculate that there may be higher parasitaemia in patients with a positive pre-treatment kDNA-PCR, resulting in slower antibody decline. No associations were found between pre- or post-treatment antibody titres and gender, age or BMI.

The Qom ethnic group had significantly higher IgG1 titre pre-treatment against lysate. Qom households receive a lower level of formal education compared to Creoles [66]. Furthermore, the Qom do not use screens or apply insecticides as regularly, and their households had a higher prevalence of domestic infestation with *T. infestans* [67] and of *T. cruzi-*infected dogs and cats [66, 68], increasing the likelihood of repeat infection by *T. cruzi* and therefore increased production of antibodies [69]. However, we do not have an explanation for the higher pre-treatment titres among the Creole community with the less discriminative IgG assay.

Limitations to this study could be addressed with a larger study cohort, more representative (broader) age distribution and additional intervening time points. Employment of qPCR may have revealed a few cases with very low parasitemia, not detected by kDNA-PCR [36]. We had insufficient pre-treatment ECG data to use in our analysis of IgG1 levels, although this would be an interesting aspect of future research. Including a cardiological and clinical evaluation may shed light on the current status of the study patients.

## Conclusion

Here, we show that IgG1 decline is more discriminative than IgG. A larger proportion of post-treatment samples showed anti-*T. cruzi* IgG1 decline in comparison to IgG, regardless of the antigen employed. Overall, post-treatment samples had significantly lower IgG1. Although IgG1 has restricted sensitivity and should not be used as a diagnostic assay, with further development it clearly has potential as a biomarker of cure. Our results emphasise the importance of early diagnosis and treatment of affected populations in endemic areas.

## Supplementary Information

**Additional file 1: Figure S1.** Timeline of field work and main activities undertaken in Pampa del Indio, Chaco, Argentina.

**Additional file 2: Figure S2.** Decline in RDT test line intensity. Examples of notable decline in Chagas Sero K-SeT test line intensity observed between pre- and post-treatment samples. a. From strong to moderate, b. From moderate to weak and c. From weak to negative. C: Control line T: Test line.

**Additional file 3: Table S1.** Multiple logistic regression analysis of antibody decline as a function of selected predictors.

**Additional file 4: Table S2.** Multiple logistic regression analysis of seronegativisation as a function of selected predictors.

**Additional file 5: Table S3.** Database.

## Data Availability

The data sets generated during and/or analysed during the current study are available as an additional file. (Additional file 5: Table S3).

## Abbreviations

BMI: Body mass index
Bz: Benznidazole
Nf: Nifurtimox
TSSA: Trypomastigote small surface antigen
TSSApep: Lineage-specific TSSA peptide
RDT: Rapid diagnostic test
