# Supplementary figures and images for: Assessing antibody decline after chemotherapy of early chronic Chagas disease patients
Source: Parasit Vectors. 2021 Oct 20;14:543. doi: 10.1186/s13071-021-05040-6 (PMC8527601; doi:10.1186/s13071-021-05040-6)

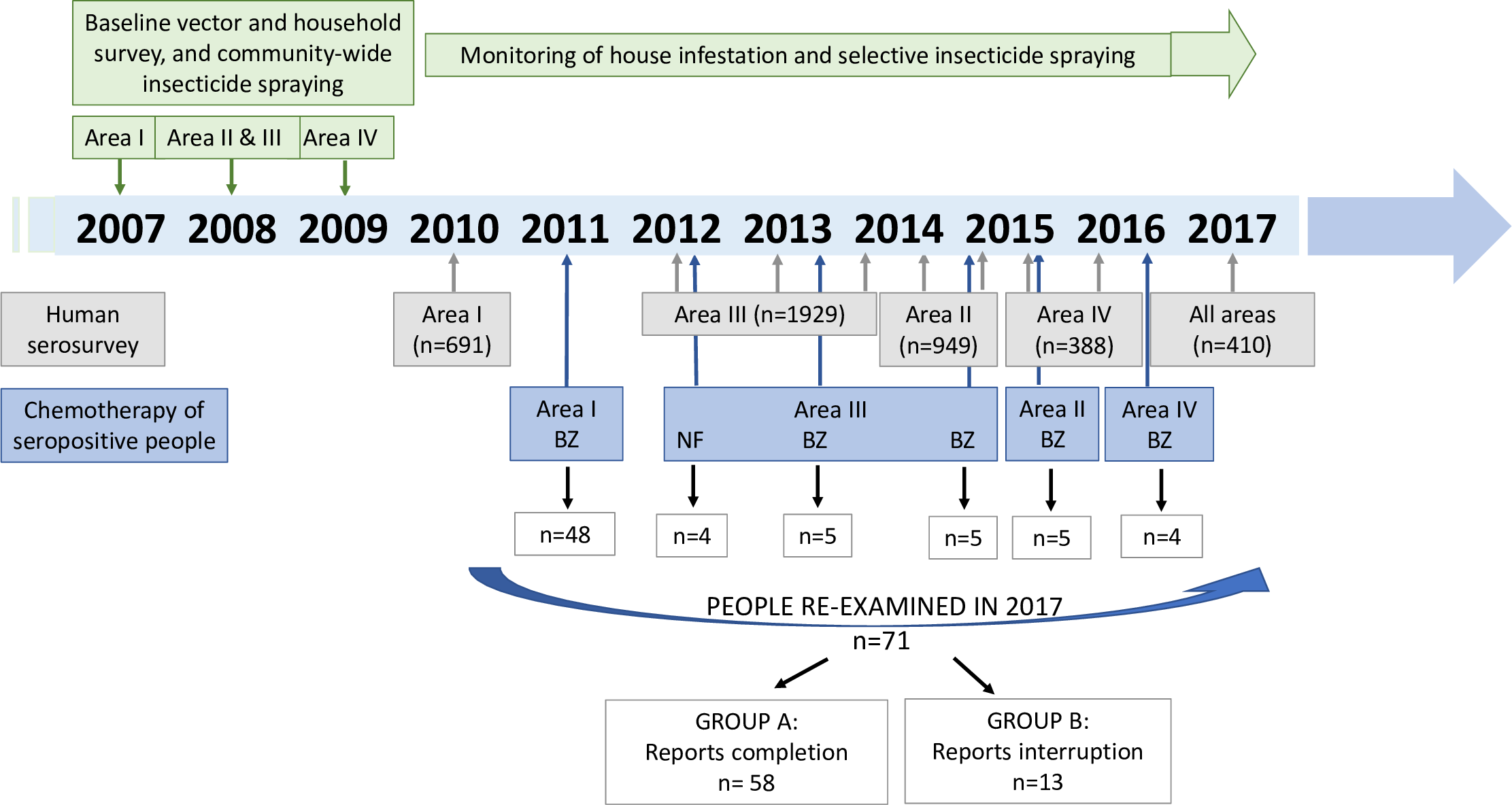

Supplement: Supplementary file 1 — Additional file 1: Figure S1. Timeline of field work and main activities undertaken in Pampa del Indio, Chaco, Argentina. [file 13071_2021_5040_MOESM1_ESM.tif]

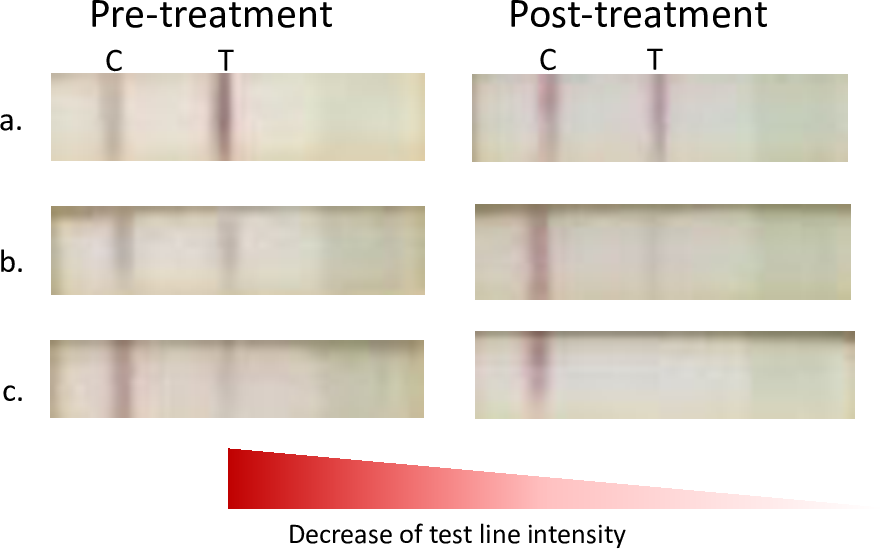

Supplement: Supplementary file 2 — Additional file 2: Figure S2. Decline in RDT test line intensity. Examples of notable decline in Chagas Sero K-SeT test line intensity observed between pre- and post-treatment samples. a. From strong to moderate, b. From moderate to weak and c. From weak to negative. C: Control line T: Test line. [file 13071_2021_5040_MOESM2_ESM.tif]
