# Supplementary material for: Assessing antibody decline after chemotherapy of early chronic Chagas disease patients
Source: Parasit Vectors. 2021 Oct 20;14:543. doi: 10.1186/s13071-021-05040-6 (PMC8527601; doi:10.1186/s13071-021-05040-6)
Supplement: Supplementary file 3 — Additional file 3: Table S1. Multiple logistic regression analysis of antibody decline as a function of selected predictors. [file 13071_2021_5040_MOESM3_ESM.docx]

Additional File 3: Table S1. Multiple logistic regression analysis of antibody decline as a function of selected predictors

|  | IgG_lysate  (n = 70) | | IgG1_lysate  (n = 57) | | IgG TSSA  (n = 51) | |
| --- | --- | --- | --- | --- | --- | --- |
| Predictors | OR | CI | OR | CI | OR | CI |
| Intercept | 0.6 | 0.2 – 2.2 | 0.9 | 0.2 – 3.9 | 0.7 | 0.2 – 2.8 |
| Age at treatment | 0.3 | 0.1 – 0.6^**^ | 0.2 | 0.1 – 0.5^**^ | 0.4 | 0.1 – 0.8^*^ |
| Treatment group | 0.7 | 0.2 – 2.7 | 5.4 | 1.0 – 34.3^*^ | 8.5 | 1.6 – 54.1^*^ |
| Time since treatment | 1.7 | 0.8 – 4.3 | 1.4 | 0.7 – 2.8 | 0.6 | 0.3 – 1.3 |

**P* ≤ 0.05, ** *P* < 0.01, *** *P* < 0.001. Abbreviations: OR, odds ratio; CI, confidence interval
