# Supplementary material for: Assessing antibody decline after chemotherapy of early chronic Chagas disease patients
Source: Parasit Vectors. 2021 Oct 20;14:543. doi: 10.1186/s13071-021-05040-6 (PMC8527601; doi:10.1186/s13071-021-05040-6)
Supplement: Supplementary file 4 — Additional file 4: Table S2. Multiple logistic regression analysis of seronegativisation as a function of selected predictors. [file 13071_2021_5040_MOESM4_ESM.docx]

Additional file 4: Table S2 Multiple logistic regression analysis of seronegativisation as a function of selected predictors

|  | IgG_lysate  (n = 70) | | IgG1_lysate  (n = 57) | | IgG TSSA  (n = 51) | | IgG1 TSSA  (n = 29) | | |
| --- | --- | --- | --- | --- | --- | --- | --- | --- | --- |
|  |  | |  | |  | |  | | |
| Predictors | OR | CI | OR | CI | OR | CI | OR | CI |  |
| Intercept | 0.1 | 0.0 – 0.4^*^ | 0.2 | 0.0 – 0.9 | 0.1 | 0.0 – 0.6^*^ | 2.4 | 0.5 – 17.2 |  |
| Age at treatment | 1.2 | 0.5 –2.6 | 0.7 | 0.3 –1.5 | 0.3 | 0.1 – 0.7^*^ | 1.0 | 0.4 – 2.7 |  |
| Treatment group | 1.3 | 0.2 – 25.9 | 3.1 | 0.7 – 22.3 | 5.1 | 1.0 –43.1 | 0.9 | 0.1 – 6.2 |  |
| Time since treatment | 1.2 | 0.5 – 3.6 | 1.4 | 0.7 – 2.8 | 0.4 | 0.2 – 0.9^*^ | 0.8 | 0.3 – 2.3 |  |

**P* ≤ 0.05, ** *P* < 0.01, *** *P* < 0.001 Abbreviations: OR, odds ratio; CI, confidence interval
